# Continuous Grading of Early Fibrosis in NAFLD Using Label-Free Imaging: A Proof-of-Concept Study

**DOI:** 10.1371/journal.pone.0147804

**Published:** 2016-01-25

**Authors:** Juho Pirhonen, Johanna Arola, Sanja Sädevirta, Panu Luukkonen, Sanna-Maria Karppinen, Taina Pihlajaniemi, Antti Isomäki, Mika Hukkanen, Hannele Yki-Järvinen, Elina Ikonen

**Affiliations:** 1 Departments of Anatomy, Faculty of Medicine, University of Helsinki, Helsinki, Finland; 2 Department of Pathology, Faculty of Medicine, University of Helsinki, Helsinki, Finland; 3 Department of Medicine, Faculty of Medicine, University of Helsinki, Helsinki, Finland; 4 Department of Pathology, HUSLAB, Helsinki, Finland; 5 Minerva Foundation Institute for Medical Research, Helsinki, Finland; 6 Faculty of Biochemistry and Molecular Medicine, Oulu Center for Cell-Matrix Research, Biocenter Oulu, Oulu, Finland; Tufts University, UNITED STATES

## Abstract

**Background and Aims:**

Early detection of fibrosis is important in identifying individuals at risk for advanced liver disease in non-alcoholic fatty liver disease (NAFLD). We tested whether second-harmonic generation (SHG) and coherent anti-Stokes Raman scattering (CARS) microscopy, detecting fibrillar collagen and fat in a label-free manner, might allow automated and sensitive quantification of early fibrosis in NAFLD.

**Methods:**

We analyzed 32 surgical biopsies from patients covering histological fibrosis stages 0–4, using multimodal label-free microscopy. Native samples were visualized by SHG and CARS imaging for detecting fibrillar collagen and fat. Furthermore, we developed a method for quantitative assessment of early fibrosis using automated analysis of SHG signals.

**Results:**

We found that the SHG mean signal intensity correlated well with fibrosis stage and the mean CARS signal intensity with liver fat. Little overlap in SHG signal intensities between fibrosis stages 0 and 1 was observed. A specific fibrillar SHG signal was detected in the liver parenchyma outside portal areas in all samples histologically classified as having no fibrosis. This signal correlated with immunohistochemical location of fibrillar collagens I and III.

**Conclusions:**

This study demonstrates that label-free SHG imaging detects fibrillar collagen deposition in NAFLD more sensitively than routine histological staging and enables observer-independent quantification of early fibrosis in NAFLD with continuous grading.

## Introduction

The subset of patients with NAFLD who have fibrosis (any stage) are at increased risk of liver-related death [1,2]. While detection of advanced fibrosis rarely constitutes a diagnostic problem, quantification of early stage(s) of fibrosis is challenging. Noninvasive techniques discriminate subjects with advanced fibrosis vs. normal liver parenchyma relatively well but are not able to accurately differentiate between minimal or moderate stages of fibrosis, for which liver biopsy remains a golden standard [3,4]. In a recent study by Bedossa et al, the same biopsy from 40 patients, who had metabolic syndrome as the only clinical risk factor for chronic liver disease, was scored by six expert liver pathologists using the recently developed SAF (steatosis, activity, fibrosis) scoring system [5]. While concordances for steatosis and activity based on the kappa index (κ) were substantial (κ = 0.61 and κ = 0.75), that for fibrosis was moderate (κ = 0.53). The major disagreement in fibrosis staging was observed amongst stage 1 subgroups. These data imply that there is a need for a sensitive, observer-independent tool to detect early fibrosis in NAFLD.

Nonlinear imaging entails several optical microscopy techniques that enable sensitive detection of native structures in cells and tissues without exogenous labels. In particular, second-harmonic generation (SHG) is a non-resonant process that takes advantage of a coherent optical signal generated by non-centrosymmetric structures, such as fibrillar collagen [6]. Coherent anti-Stokes Raman scattering (CARS), on the other hand, produces images based on characteristic intrinsic vibrational frequencies of chemical bonds. Strong resonant CARS signals are generated from lipids due to their abundant CH_2_ hydro-carbon bonds [7,8]. Thus, SHG and CARS imaging can be employed for sensitive detection of fibrosis and fat from tissues in a label-free fashion. The potential of SHG imaging to detect human liver fibrosis has been examined in patients with fibrosis due to hepatic B and C infection [9,10]. In Gailhouste et al, the SHG signal was closely correlated with the intensity of fibrillar collagen I and III stainings and the Metavir fibrosis score. In both studies, the authors developed methods for scoring the SHG signal, which was suggested to allow reproducible quantification of fibrosis independently of operators [9,10].

In NAFLD, fat initially accumulates around the central vein in zone 3. In this area, which has lower levels of oxygen than the periportal area [11], hepatocytes undergo ballooning necrosis and stellate cells become activated in the perisinusoidal areas, leading to fibrogenesis. Fibrosis develops as an imbalance between extracellular matrix deposition and degradation. How these dynamic changes are reflected in clinical disease outcome, is uncertain [12]. Moreover, there are disease-specific pathways of fibrosis, and fibrotic changes in fatty liver are distinct from those in hepatitis, which is initially observed in portal areas [12,13]. This emphasizes the need to develop distinct scoring systems for fibrosis in chronic viral hepatitis and NAFLD [14].

Until now, there are no data available on the utility of SHG imaging in NAFLD. In the present proof-of concept study, we wished to determine whether it is possible to develop an automated analysis of liver fibrosis in NAFLD and whether the use of SHG imaging might be able to detect early, subtle signs of fibrosis better than routine histopathology.

## Materials and Methods

### Study subjects

The patients underwent a metabolic study for clinical characterization approximately one week prior to the liver biopsy, which was taken during bariatric surgery. Subjects were eligible if they met the following criteria: (a) age 18 to 75 years; (b) no known acute or chronic disease except for obesity or type 2 diabetes on the basis of medical history, physical examination and standard laboratory tests (blood counts, serum creatinine, electrolyte concentrations); (c) alcohol consumption less than 20 g per day and less than 30 g for men; (d) no clinical or laboratory evidence of other liver disease. Patients were excluded if they were pregnant. The study protocol was approved by the ethics committee of the Helsinki University Central Hospital and follows the 1975 Declaration of Helsinki guidelines. Each participant provided written informed consent. At the metabolic visit, a blood sample was taken after an overnight fast for the screening laboratory tests (vide supra) as well as for measurement of fasting plasma glucose, serum insulin, fS-LDL cholesterol, total serum cholesterol, fS-HDL cholesterol, fS-triglycerides, fS-AST, fS-ALT, and fS-GGT concentrations as described [15]. Body weight and height, waist and hip circumferences, blood pressure and the electrocardiogram were recorded as described [15].

### Liver biopsies

Wedge biopsies of the liver (50–150 mg) were taken at laparoscopic surgery. Approximately one-half of the liver sample was fixed in formalin and sent to the pathologist, who was unaware of the SHG-scoring data, for histopathological assessment, whereas the rest was immediately frozen and stored in liquid nitrogen. The sample frozen in liquid nitrogen was embedded in optimal cutting temperature (OCT) compound and cryosectioned at 20μm thickness. Slices were fixed with 4% paraformaldehyde for 60 min and a coverslip added. The prepared slides were stored at -20°C for no longer than a week prior to examination by non-linear microscopy.

### Histological assessment

NASH was defined according to the scoring system proposed by Kleiner et al. [14]. For histologic analysis, tissue sections were stained with hematoxylin and eosin, impregnated with silver for reticulin framework, and stained with trichrome for collagen. All biopsy samples were representative and most of them had more than 20 portal tracts (a minimum of 6 portal tracts). The amount of steatosis, inflammation and fibrosis was analyzed. The percentage of steatotic hepatocytes containing micro- and macrovesicular fat was scored. Inflammatory activity (included foamy degeneration of hepatocytes, sinusoidal fibrosis and neutrophil infiltration) of steatohepatitis was scored from 0–3. The stage of fibrosis was scored from 0–4: (0: no fibrosis, 1: portal or sinusoidal fibrosis without bridging septa, 2: portal or sinusoidal fibrosis with few bridging septa, 3: advanced fibrosis with numerous septa, and 4: fully developed cirrhosis). Any overlap pathology was ruled out. Every sample was assessed by two pathologists, the clinical pathologist making the original histological diagnosis and the research pathologist independently re-evaluating the samples. Their inter-rater agreement for fibrosis stage was 0.39.

### Non-linear microscopy

Images were acquired with a commercial Leica TCS SP8 CARS confocal microscope. The instrument consists of an inverted microscope equipped with an ultra-short pulsed light source (picoEmerald, APE, Berlin, Germany) that produces the two synchronous beams needed for CARS microscopy. The Stokes beam at 1064 nm was emitted from a neodymium-doped yttrium orthovanadate (Nd:YVO4) laser while a tunable pump/probe beam at 780–940 nm was generated by an optical parametric oscillator (OPO). The pulse width was 5–7 ps with a repetition rate of 80MHz corresponding to the Raman line width of 2–3 cm^-1^. The pulses from the two sources were temporally and spatially overlapped on the focal plane of the microscope. Up to 100 mW of average power from both the pump and the Stokes source was delivered to the sample. No signs of photodamage as assessed in [16] were observed with these scanning parameters. All samples were imaged with identical laser intensity and previously imaged samples were used as a reference. The generated SHG and CARS signals passed through suitable bandpass filters and were detected in the forward-direction using a non-descanned photomultiplier tube (PMT) detector. For SHG imaging the laser was set up at a wavelength of 816.5nm. The same laser was used for the CARS modality simultaneously with the Stokes beam at 1064 nm to excite the symmetric vibrational resonance of the CH_2_ hydro-carbon bonds at 2845 cm^-1^. Images were acquired from unstained slides using a 25x water immersion objective (Leica HCX IR APO L 25X/0.95 W). To cover the area of the entire biopsy specimen (about 4 x 4 mm), multi-tile scanning (up to 25x25 tiles) was performed. All images were recorded using the Leica Application Suite Advanced Fluorescence (LAS AF) software.

### Image analysis

The images were processed and analyzed using Fiji [17]. Background signal, determined as mean signal intensity outside of the sample, was subtracted, after which the sample mean SHG-intensity was measured (portal areas and capsule excluded). An algorithm using iterations of mean filtering, thresholding and particle analysis was developed to recognize and exclude the capsule and portal areas from the measurements (see below for details). Image analysis was divided into three parts: A) determining sample borders, B) determining the portal areas, C) measuring signal intensities.

1. Determining sample borders: The signals acquired from the SHG and CARS channels were overlayed to highlight the sample area. The image was then auto-thresholded by the percentile method [18] and converted to a binary mask. Iterations of filtering were then performed to smoothen the image (mean, maximum and mean filtering with radii of 50μm, 15μm and 50μm). This 8-bit image was then thresholded (9.8% of maximum pixel intensity) to yield the final area.
2. Determining portal areas: The SHG channel was extracted from the image. To remove background, the 32-bit image was auto-thresholded to mean intensity. Next, iterations of filtering were used to harmonize the highly fibrillar structure of collagen fibrils (mean, maximum and mean filtering with radii 15μm, 35μm and 25μm). Finally, the image was thresholded (1.17% of maximum pixel intensity) for particle analysis. Recognized particles were filtered with loose size and roundness parameters.
3. Measuring signal intensities: The background signal of the original image was first removed, by subtracting the mean signal intensity outside of the sample (recognized in step A). Mean SHG- and CARS-signal intensities were then measured after excluding the portal areas and the capsule.

### Immunofluorescence microscopy

Human tissue samples were analyzed using a basic immunofluorescence protocol for formalin-fixed and paraffin-embedded tissue sections. In brief, 5-μm sections underwent xylene/ethanol rehydration series and were blocked with 1% BSA in PBS (pH 7.2) for 1h, incubated with a primary antibody overnight at 4°C and with the secondary antibody for 1 hour at room temperature. All washes were performed with PBS. The following primary antibodies were used with the dilutions indicated: collagen I (600-401-103, Rockland Immunochemicals, Limerick, PA, USA, 1:800) and collagen III (600-401-105, Rockland Immunochemicals, 1:400). Before staining, epitope retrieval was performed by a heat-mediated method for 15 min with sodium citrate buffer (pH 6.0). Anti-rabbit Cy3 (Jackson Immunoresearch, West Grove, PA, USA, 1:300) was used as a secondary antibody. For negative control stainings, the primary antibodies were omitted and replaced by PBS. Immunostaining with different antibodies was performed on parallel sections.

### Statistics

For the patient data, continuous variables were tested for normality using the Kolmogorov-Smirnov test. Normally distributed data were reported in means ± SEM while non-normally distributed were reported in medians and interquartile ranges. For the microscopy data, the Mann-Whitney-U test was used to estimate statistical significance. Inter-rater agreement was evaluated using the weighted kappa score. Statistical analyses were performed using IBM SPSS Statistics 22.0.0.0 version (IBM, Armonk, NY).

## Results

### Patients

A total of 32 patients were studied, of whom 11 were men and 21 were women. The mean BMI of the subjects was 47.2 ± 1.0 kg/m2 and the median age 47.5 (41.5–58.0) years. Their key laboratory findings are summarized in **Table 1**. In liver histology, the median proportion of hepatocytes with macrovesicular steatosis averaged 20 (10–39) %. The distribution of fibrosis stage (0/1/2/3/4) among the subjects was: 12 (37.5%), 11 (34.4%), 5 (15.6%), 3 (9.4%) and 1 (0.03%); and for steatosis grade (0/1/2/3): 19 (59.4%), 8 (25%), 3 (9.4%) and 2 (6.3%).

**Table 1:** Key laboratory findings of the study subjects.

Total	All subjects (n = 32)
fP-Glucose (mmol/l)	6.1 (5.5–6.8)
fS-Insulin (mU/l)	16.3 ± 1.5
fP-Total cholesterol (mmol/l)	4.0 ± 0.2
fP-Triglycerides (mmol/l)	1.46 ± 0.1
fP-HDL cholesterol (mmol/l)	1.20 ± 0.1
fP-LDL cholesterol (mmol/l)	2.3 ± 0.1
P-AST (IU/l)	32 (27–40)
P-ALT (IU/l)	34 (27–52)
P-GGT (U/l)	30 (20–58)

Data are in means ± SEM or median (25th-75th percentile), as appropriate.

### Sample preparation and multimodal label-free imaging of liver biopsies

**Fig 1:**
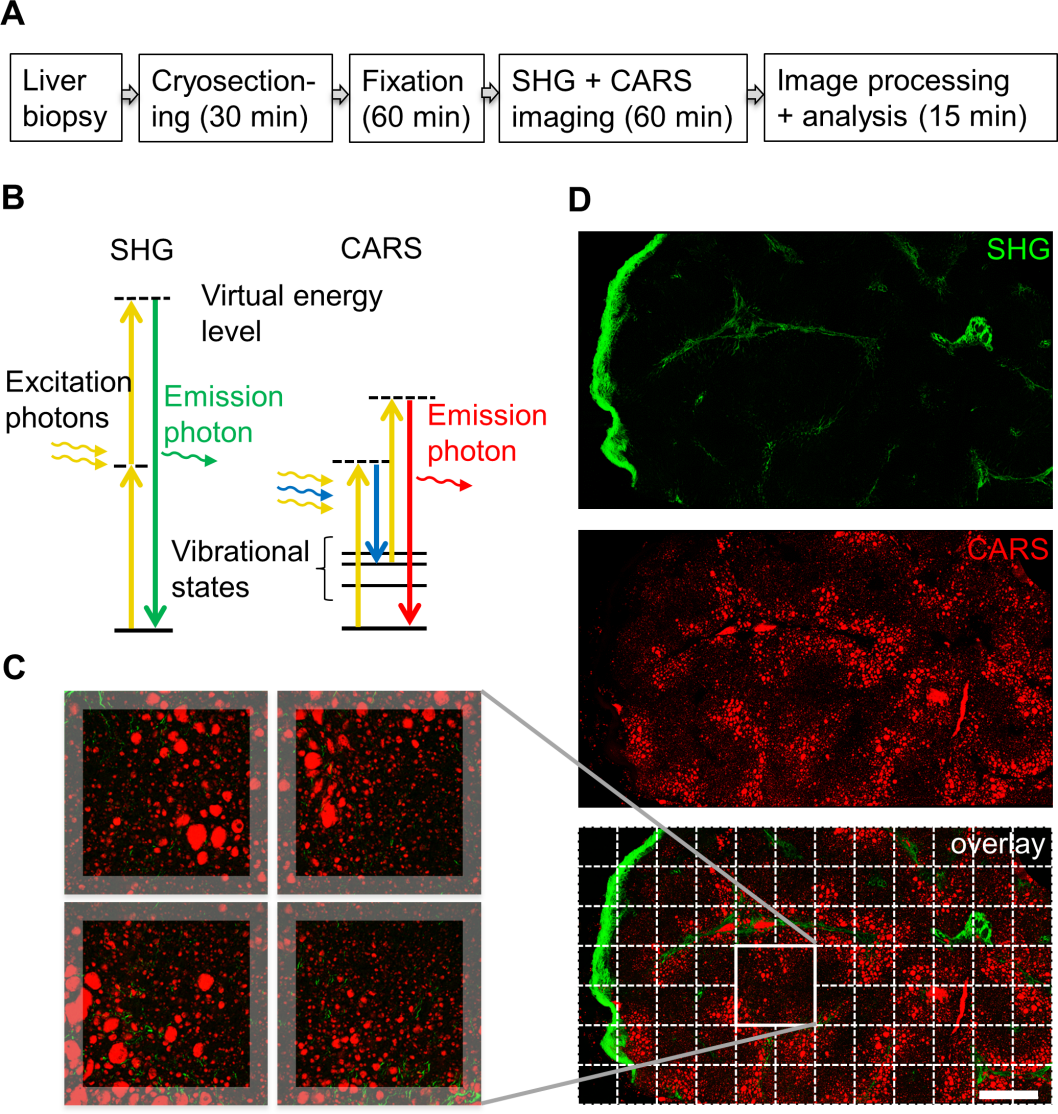
Sample processing steps, SHG and CARS imaging of NAFLD biopsy. A. Workflow and estimated time required for analysis steps. B. Schematic diagram of the SHG and CARS principles. In the SHG process, the energy of two pump photons (yellow arrows) is combined to produce one emission photon (green arrow). In the CARS process, the pump (yellow arrows) and the Stokes laser (blue arrow) stimulate a selected molecular vibration. Probing of this vibration with the second pump photon results in the CARS emission (red arrow). Dotted lines represent virtual states of excitation. C. Biopsy reconstruction from stitched images. D. Simultaneous SHG and CARS imaging of a NAFLD biopsy, separate channels and the overlay are shown. Scale bar 500um.

An overview of the preparation of biopsies for SHG and CARS imaging and the timeline is shown in **Fig 1A**. Freshly frozen biopsies were cryosectioned, followed by immediate mild fixation of the sections in 4% paraformaldehyde, to minimize sample manipulation. Imaging was performed on a confocal microscope capable of both SHG and CARS imaging, using near-infrared laser excitation at 816.4 nm and 1064.5 nm, and taking advantage of the characteristic changes in the photon energy states (**Fig 1B**). The entire biopsy (typically about 4x4 mm) was imaged, with up to 25 x 25 overlapping squares and the images stitched together (**Fig 1C**). An example of a mosaic image of a biopsy after simultaneous recording of SHG and CARS signals and reconstruction of the data set to display the entire specimen is shown in **Fig 1D**.

### Relationship between histologic fibrosis stage with the SHG and steatosis grade with the CARS signal

**Fig 2:**
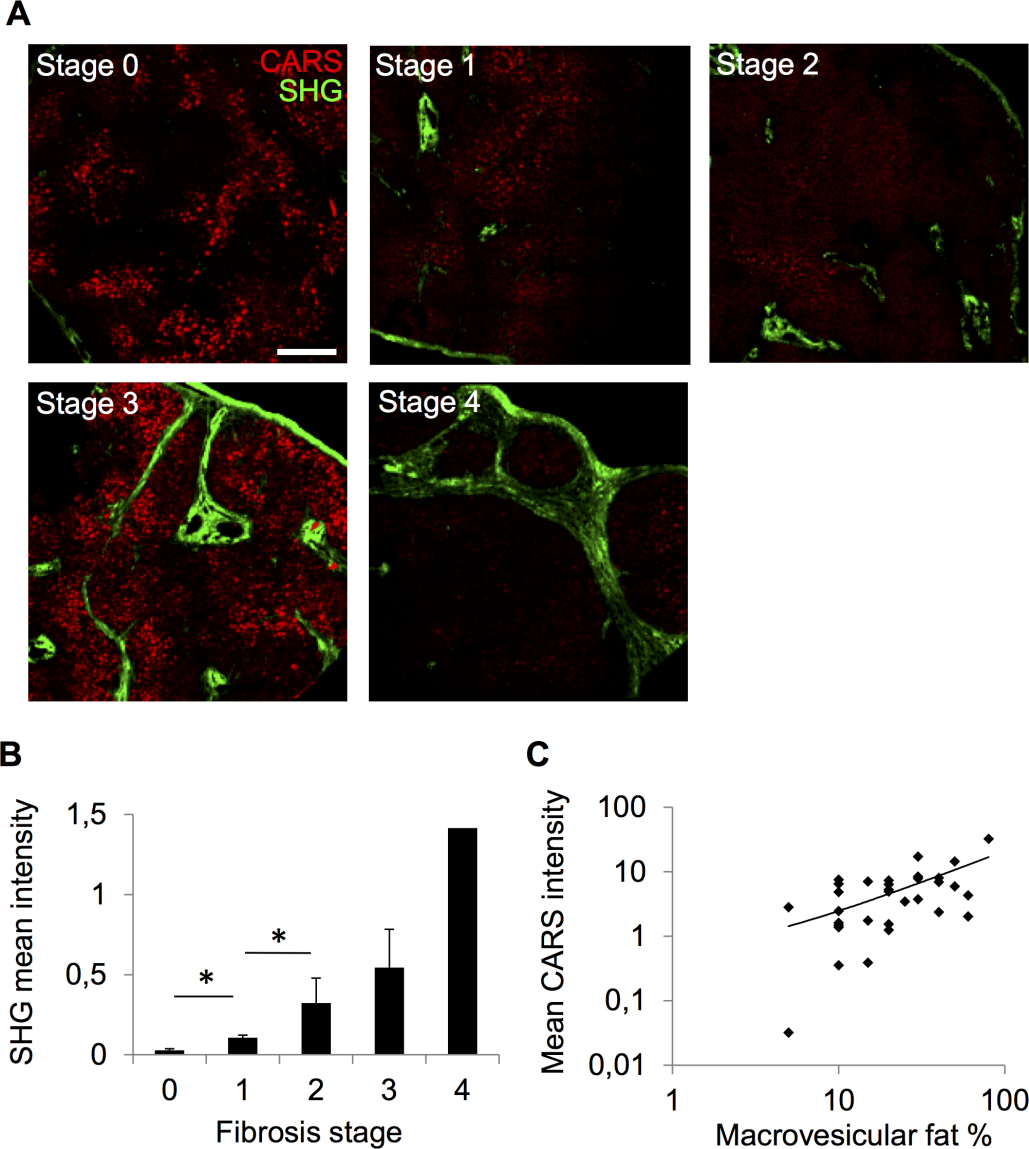
Correlation of SHG intensity with liver fibrosis and CARS intensity with liver fat. A. Exemplary SHG and CARS images of fibrosis stages 0–4. Scale bar 500μm. B. SHG mean intensity in samples representing different fibrosis stages, stage 0 n = 12, stage 1 n = 12, stage 2 n = 4, stage 3 n = 3, stage 4 n = 1, *p<0.05 between stages 0 and 1 and between 1 and 2. C. CARS mean intensity in samples (n = 32) representing different amounts of macrovesicular fat as assessed by pathologist; Spearman’s rank correlation coefficient 0.504, p<0.005.

To analyze if the SHG signal intensity correlated with histological fibrosis staging, we analyzed 32 samples, from which parallel samples had been histologically evaluated by a pathologist. As the patients were undergoing bariatric surgery, advanced stages of fibrosis (3 and 4) were rare in this material. Examples of SHG images of stage 0–4 fibrosis are shown in **Fig 2A**. In stages 1 and 2, the signal from the portal areas typically predominated, while in stages 3 and 4, marked signals were also observed in septal regions, in agreement with the expected distribution of fibrosis in these stages. Moreover, there was a good overall correlation between the mean intensity of the SHG signal and fibrosis stage in all samples (**Fig 2B**). In the same samples, the mean signal intensity of the CARS signal correlated positively with histologically determined macroscopic steatosis (**Fig 2C**).

### Development of automated image analysis for early fibrosis

We next developed an automated image analysis method to evaluate and quantify early liver fibrosis from the biopsies using SHG. To this end, we excluded SHG signals derived from the capsule and from portal areas. This is because these regions generate a prominent SHG signal even in a normal liver, due to their physiologically high collagen content, and this masks any weaker signal from elsewhere in the parenchyma. Furthermore, the area occupied by portal regions and the capsule varied considerably between biopsies, affecting signal mean intensity. The automatic procedure developed for exclusion of these areas involved background subtraction and thresholding, filtering, particle identification and exclusion, prior to SHG and CARS intensity measurements in the remaining area, is illustrated in **S1 Fig**.

### Detection and characterization of early parenchymal fibrosis using SHG imaging

**Fig 3:**
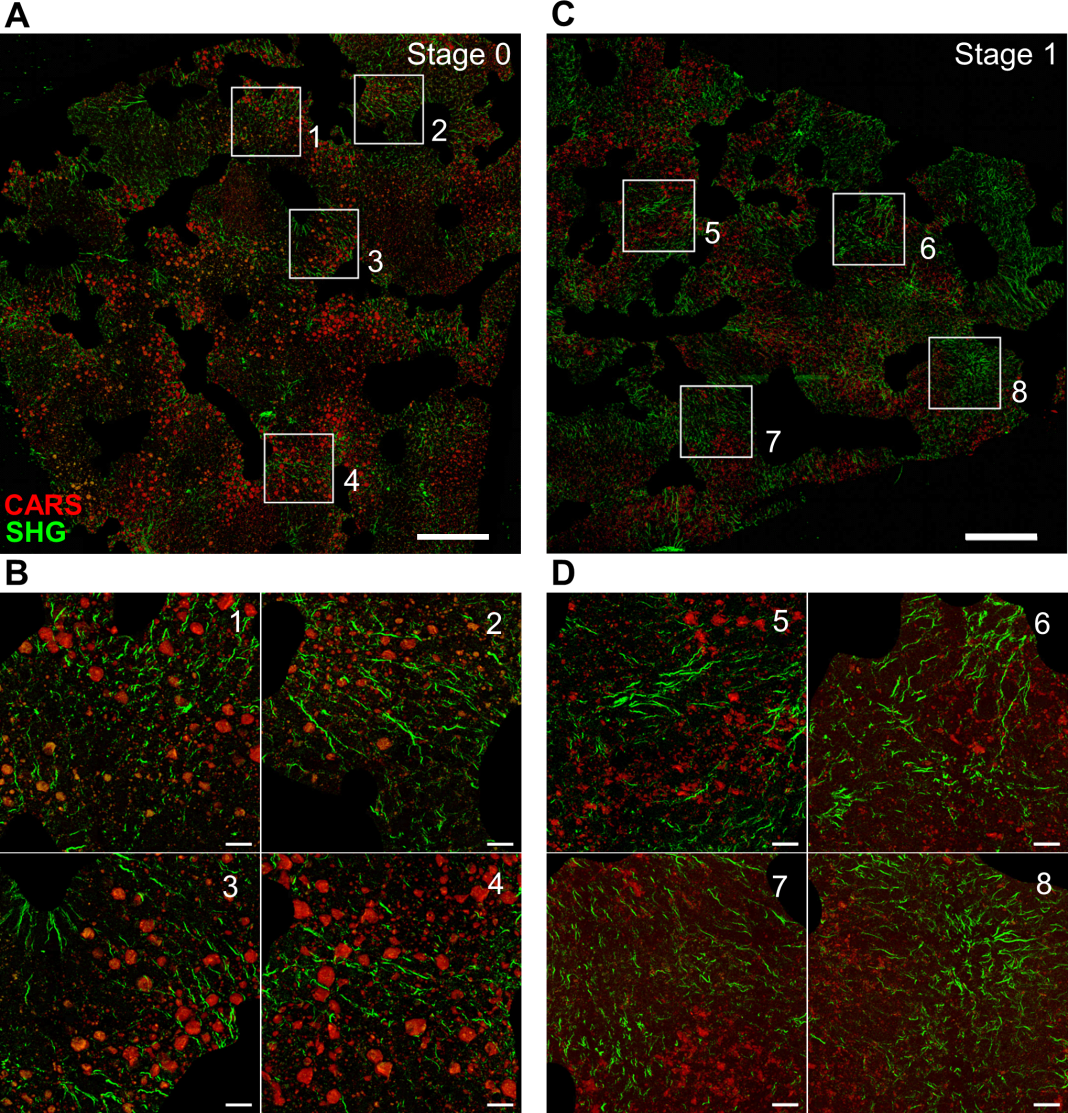
High-resolution imaging of fibrillar and lipid signals in NAFDL stage 0 and stage 1 fibrosis. A. Stage 0 fibrosis, B. Higher magnification insets (1–4) shown as white rectangles in A. C. Stage 1 fibrosis. D. Higher magnification insets (5–8) shown as white rectangles in C. Scale bar 500μm in A and C, and 50μm in B and D.

The SHG signal derived from the liver parenchyma and included in the analysis was fibrillar in nature (**Fig 3**). Remarkably, we found that already stage 0 NAFLD samples showed specific, albeit weak, fibrillar SHG signals. These were typically relatively short (20–200 micron length) wavy fibrils or narrow bundles of fibrils (**Fig 3A and 3B**). In stage 1, the signal was essentially similar in character but these thin threads were more densely distributed between cords of hepatocytes, resulting in an overall higher SHG signal (**Fig 3C and 3D**). The fibrils often appeared to bridge between portal areas (excluded from the analysis, black regions within tissue section in **Fig 3**), reminiscent of early septal fibrosis (**Fig 3B,** insets 2 and 3). The fibrils were interspersed between heterogeneously sized lipid deposits as visualized by the CARS signal. This signal derives from lipid-laden hepatocytes.

**Fig 4:**
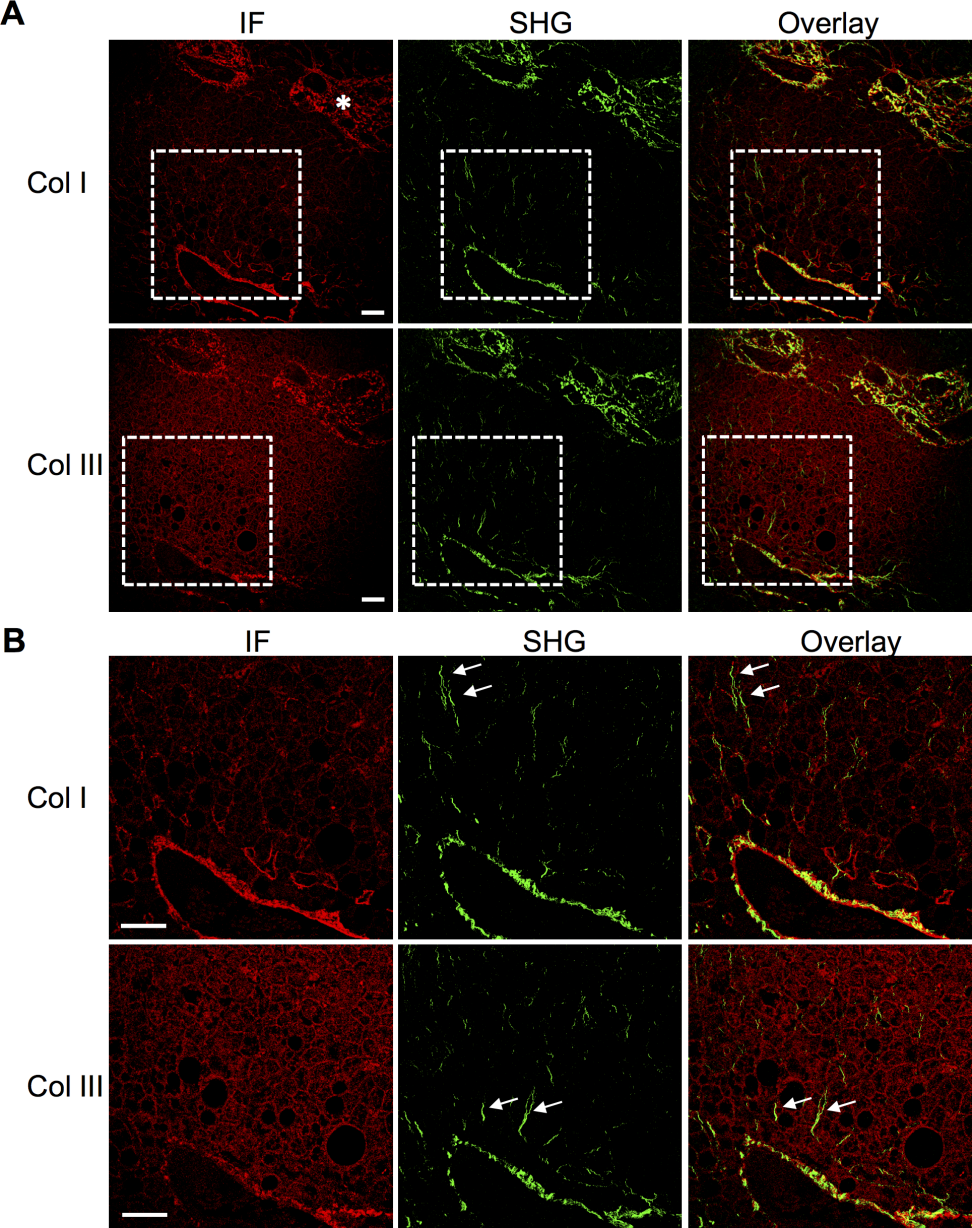
Immunofluorescence and SHG detection of collagens in NAFLD stage 0 fibrosis. Immunofluorescence (IF) stainings of collagens type I and III displayed as red and SHG signals from the same sections displayed as green, colocalization is displayed as yellow in the overlay image. White rectangle in A shows the position of insets highlighted in B. Asterisk indicates a portal triad. Arrows indicate examples of fine SHG signals not evident by collagen immunostaining. Scale bar: 50μm.

To assess if the SHG signal in early fibrosis is specific and derives from collagens, we performed indirect immunofluorescence stainings of stage 0 fibrosis NAFLD samples using anti-collagen antibodies. The samples were imaged using both fluorescence and SHG channels. Due to solvent exposure during processing, lipids were extracted in these samples and lipid-rich areas appeared as holes in the specimen. We found specific immunoreactivity for the major fibrillar collagens I and III, with prominent labelling of collagen I in the extracellular matrix surrounding the central vein and the portal area (**Fig 4A**). The collagen III signal concentrated in the same areas but was weaker, presumably due to lower abundance of collagen III and/or weaker antibody affinity. Importantly, both collagen signals overlapped with the SHG signal in the same areas (**Fig 4**), demonstrating specificity of the SHG signal. Moreover, SHG imaging revealed multiple thin threads that emanated from the central vein but were not highlighted by the antibody stainings (**Fig 4B**), suggesting that SHG microscopy is more sensitive than collagen immunostaining in detecting early fibrosis.

### SHG imaging reveals positive signals in stage 0 fibrosis

**Fig 5:**
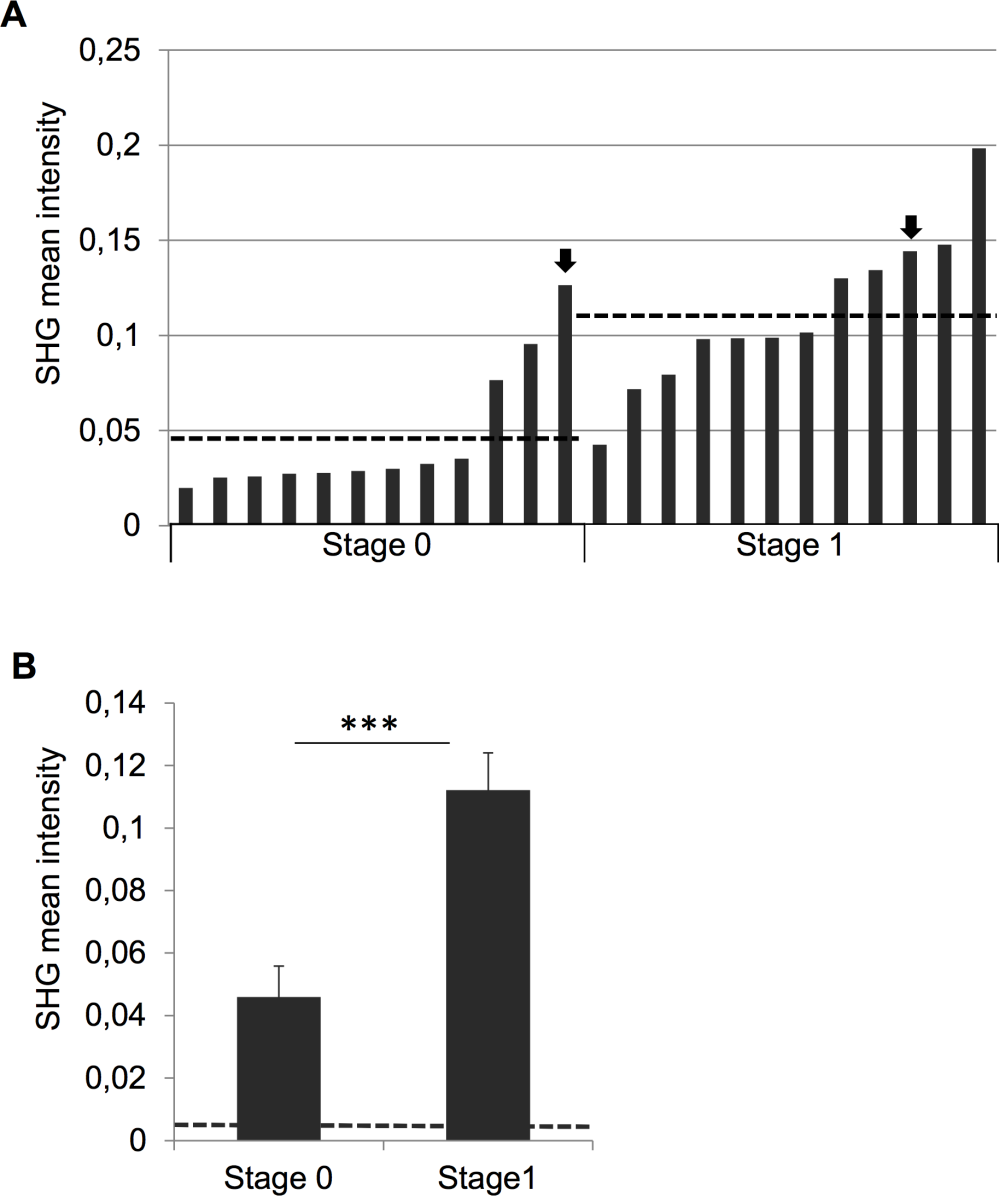
SHG imaging provides quantitative assessment and detects fibrosis in stage 0 NAFLD. Mean SHG intensities from individual NAFLD biopsies analyzed using automated signal analysis. In A, arrows indicate the F0 and F1 samples shown in Fig 3 and dashed lines show average intensities of stage 0 and 1 samples. B. Comparison of SHG mean intensity in stage 0 and 1 fibrosis. Dashed line indicates average background SHG signal intensity from outside the sample area. ***p<0.001.

We examined whether the automated SHG imaging system can be used for detection of early fibrosis and whether it correlates with the stage of fibrosis as determined by histology. For this, we compared the SHG signal from 24 NAFLD samples with the degree of fibrosis (stage 0 or 1). This analysis showed that on average, stage 1 samples had significantly higher SHG intensities than stage 0 samples (**Fig 5A and 5B**), indicating that SHG imaging can differentiate between these stages of fibrosis. SHG imaging showed a roughly 5-fold difference in signal mean intensity within the stage 1 samples (**Fig 5A**). Three stage 0 samples (independently scored as stage 0 fibrosis by two pathologists) had higher SHG intensities than the lowest stage 1 sample, and one of them had a higher intensity than stage 1 samples on average (**Fig 5A**). The average SHG signal intensity in stage 0 fibrosis samples was approximately 8-fold above background (**Fig 5B**). This suggests that SHG imaging can detect early fibrosis in NAFLD more sensitively than routine histological staging.

## Discussion

In this study, we tested the capability of a recently established label-free imaging modality, SHG microscopy, in assessing the initial stages of fibrosis in NAFLD. Our findings provide evidence that SHG imaging can detect early deposition of fibrillar compounds better than routine histopathology. SHG seems to improve the detection sensitivity for very fine fibrillary structures, apparently representing the earliest signs of fibrosis, and enables quantitative assessment of these signals with continuous grading. We also demonstrate the capability of a new, in-house developed automated image analysis platform in providing observer-independent quantification of early fibrosis. This is relevant as there is considerable inter-rater disagreement in fibrosis staging, especially in early stages of fibrosis in NAFLD [5].

SHG imaging has previously been used to quantify liver fibrosis in patients with hepatitis B and C [9,10]. Gailhouste et al. [9] developed a quantitative SHG scoring method that was particularly suited for assessing advanced fibrosis. They demonstrated the capability of SHG microscopy in discriminating advanced fibrosis and cirrhosis. Instead, in non-advanced (Metavir F0-F1) fibrosis, the SHG indices overlapped. The authors also reported a good relationship between SHG signal and collagens over-produced during fibrosis progression, in agreement with our study. Recently, Xu et al. [10] developed another SHG based scoring method that differentiated between Metavir stages F1-4 in chronic hepatitis B. This comprised 12 samples with F1 and 9 with F2 fibrosis; however, F0 samples were not included in this cohort.

The present report provides, to our knowledge, the first assessment of early fibrosis in NAFLD using SHG imaging. It is important to note that the location and distribution of fibrosis—and thereby SHG signal generation—depends on the etiology of liver disease. In chronic hepatitis, the inflammatory activity is typically dominating in the interface area of portal tracts and lobules, inducing fibrosis that is primarily periportal [13]. Instead, in steatohepatitis the overall location of the degenerative process is different: NASH activity is mainly located in the pericentral region and activated stellate cells play a major role in forming the characteristic sinusoidal fibrosis [19–21].

In normal liver, several of the major collagens, such as IV and VI, are non-fibrillar and therefore not expected to generate prominent SHG signals. Instead, the major fibrillar collagens, types I and III, are expressed upon development of liver fibrosis. Their amounts increase in NAFLD related fibrosis [22,23] and give raise to SHG signals [24], as demonstrated in the present study. Of the other fibrillar collagens expressed in the liver, type V collagen is considerably less abundant and assembles with type I and III collagens to form composite, heterotypic fibrils. When fibrosis advances to cirrhosis, all these fibrillar collagen types increase significantly [25].

SHG imaging offers a number of advantages in comparison to traditional histological assessment of fibrosis. Firstly, sample preparation is fast as it does not require de-waxing or other lengthy preparation steps. Secondly, it provides sensitive, quantitative and operator-independent assessment of fibrosis that can be automated. This enables reliable monitoring of incipient extracellular matrix deposition, which has until now been difficult to reliably quantify. Thirdly, SHG imaging requires no staining and directly detects fibrillar supramolecular structures, due to their inherent physical properties of non-centrosymmetry and high crystalline structure [24]. This is in contrast to traditional histological stains used for the detection of extracellular matrix, including collagen, and based on indirect methods (such as acidity, properties of incorporated contrasting dyes or metal ions). Finally, combination of SHG with other non-linear and linear optical modalities is straightforward, as demonstrated here by combining SHG with CARS or immunofluorescence microscopy. SHG imaging also has some limitations: commercial instruments are rather costly and require a skilled operator to set up. However, prices tend to go down once techniques become more routine and once established, SHG imaging is relatively simple to perform.

In the future, the automated SHG quantification method developed should be validated in additional patient cohorts. Particularly relevant are samples with mimimal or no histochemically detectable fibrosis, for which this method might provide added value. It would also be interesting to adopt SHG imaging for the examination of repeated liver biopsies, in combination with multimodal follow-up of fat deposition by CARS and inflammatory cell accumulation e.g. by immunofluorescence detection. Where paired biopsies are available, SHG imaging has significant potential for improved assessment of fibrosis progression or regression compared to currently employed techniques. With increased sensitivity and quantitativeness for fibrosis assessment, SHG imaging should help to better define the dynamic nature of fibrosis, including the efficacy of drug or other therapy responses.

In conclusion, this study demonstrates that label-free SHG imaging enables observer-independent quantitative detection of early fibrosis in NAFLD with continuous grading. Importantly, SHG imaging was more sensitive than routine histological staging in detecting the early fibrotic processes in NAFLD. This feasibility study provides a basis for employing this method in larger NAFLD patient cohorts.

## Supporting Information

S1 Fig Overview of automated image analysis procedure. Exemplary images of analysis steps. A. Overlay of background subtracted CARS and SHG images. B. Filtered image. C. Filtered areas for exclusion. D. Final image for analysis. White line indicates the sample area and green lines excluded portal areas.
(TIF)
