# Supplementary figures and images for: Continuous Grading of Early Fibrosis in NAFLD Using Label-Free Imaging: A Proof-of-Concept Study
Source: PLoS One. 2016 Jan 25;11(1):e0147804. doi: 10.1371/journal.pone.0147804 (PMC4726624; doi:10.1371/journal.pone.0147804)

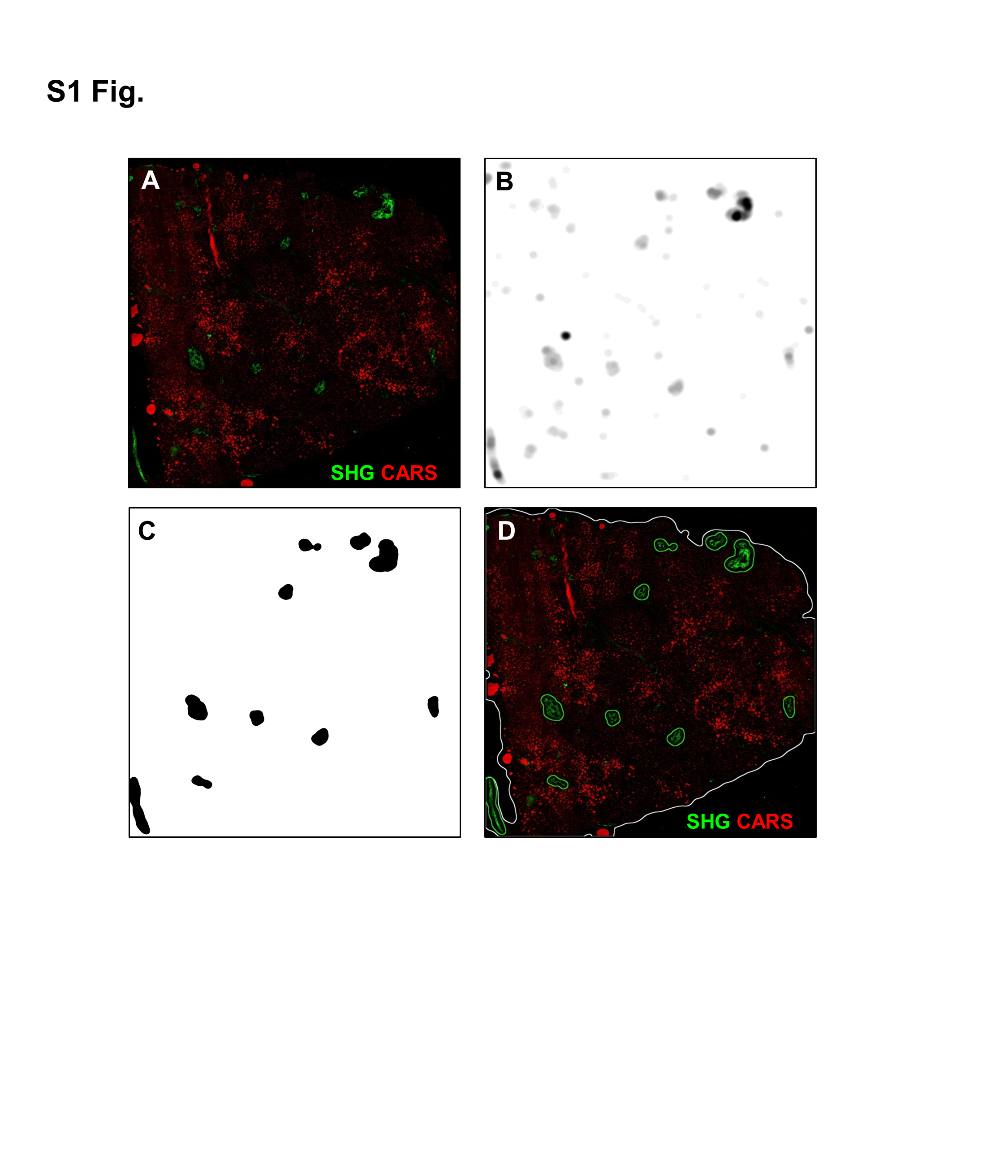

Supplement: S1 Fig — Exemplary images of analysis steps. A. Overlay of background subtracted CARS and SHG images. B. Filtered image. C. Filtered areas for exclusion. D. Final image for analysis. White line indicates the sample area and green lines excluded portal areas. (TIF) [file pone.0147804.s001.tif]
